# The role of total polysaccharides from *Sonchus arvensis L.* in the prevention and treatment of colitis via regulation of gut microbiota and metabolites

**DOI:** 10.3389/fphar.2025.1657918

**Published:** 2026-01-12

**Authors:** Yachao Ren, Shenghua Hou, Guoyou Chen, Yulong Zhou

**Affiliations:** 1 College of Animal Science and Technology, Heilongjiang Bayi Agricultural University, Daqing, China; 2 Harbin Medical University-Daqing, Daqing, China

**Keywords:** *Sonchus arvensis* L., polysaccharide, colitis, gut microbiota, non-targeted metabolomics analysis

## Abstract

**Background:**

*Sonchus arvensis* L. is a traditional Chinese food and medicine, and the primary plant metabolites are polysaccharides (SAP). In this study, we aimed to investigate the protective effect of SAP in a colitis model and the potential underlying molecular mechanisms.

**Methods:**

C57BL/6 mice were randomly assigned to three groups: the negative control, model, and SAP treatment groups. The influence of SAP on ulcerative colitis (UC) was evaluated by water and food intake, body weight change, diarrhea, bloody stool, colon length, histological analysis, disease activity index, and immune parameters. The effects of SAP on the gut microbiota (GM) were investigated using 16S rRNA sequencing. The impact of SAP on metabolites was evaluated using non-targeted metabolomics analysis.

**Results:**

SAP attenuated colitis and modified immune parameters. 16S rRNA sequencing showed that the abundance of *Akkermansia*, *Rikenella*, Rikenellaceae_RC9_gut_group, and unidentified_Clostridia_vadinBB60_group in the model mice was remarkably reversed after SAP treatment. The correlation analysis of GM and the metabolites showed that SAP could regulate five metabolites correlated with GM.

**Conclusion:**

The protective effect of SAP on the model mice may be related to GM diversity and metabolites.

## 1 Introduction

Digestive system diseases can severely impact the patients’ ability to study, work, and carry out daily life activities (Guindi and Riddell, 2004). Ulcerative colitis (UC) is a chronic inflammatory disease of the gastrointestinal tract (Seoane-Viaño et al., 2021; Khor et al., 2011) that is prone to relapses and is difficult to treat. Numerous reports suggest that the incidence and prevalence of UC have been increasing globally over the past few decades (Molodecky et al., 2012). In addition to the developed countries, recent studies in newly industrialized countries have demonstrated that UC prevalence has also increased in Asia, Africa, and South America (Kaplan and Ngm, 2017). Epidemiological findings have shown that the severity and duration of UC also increase the risk of colon cancer in patients (Cosnes et al., 2011; Terzic et al., 2010).

The primary pathological mechanisms underlying UC remain unknown. The specific etiology of UC involves multiple factors, including genes, immune function, intestinal microbiota, lifestyle, and environmental factors. Several studies showed that dysbiosis of the gut microbiota (GM) is associated with inflammatory bowel disease (IBD) (Nishida et al., 2018). Therefore, GM dysbiosis is considered the primary pathological mechanism of UC. Increasing evidence suggests that the microbiota composition of UC patients differs from that of healthy people (Andoh et al., 2007; Fujimoto et al., 2013; Sartor and Wu, 2017; Takahashi et al., 2016). Biodiversity and species richness in UC patients have decreased (Nishida et al., 2018).

Furthermore, changes in bacterial composition may alter the metabolite levels, which may contribute to the specific etiology of UC. Metabolomics analysis has revealed a correlation between bacterial composition and metabolic pathways (Wishart et al., 2007). Changes in bacterial metabolic pathways occur in approximately 12% of individuals compared with healthy individuals.

Many studies have indicated that dietary polysaccharides can modulate the gut microbiota (Cai et al., 2019). Polysaccharides are one of the four fundamental biomolecules of life and are natural biological macromolecules widely found in microorganisms, plants, and animals. Many polysaccharides and their derivatives have been reported to possess critical bioactive functions, such as anti-inflammatory, anti-aging, anti-oxidation, antiviral, anti-radiation, anti-tumor, anti-thrombosis, and hypolipidemic effects; immunoregulation; and regulation of the gut microbial composition (Miani et al., 2004; Kouakou et al., 2013; Wu et al., 2014; Bahorun et al., 2003; Kao et al., 2005). Polysaccharides have attracted significant attention from researchers due to their remarkable properties, including prominent pharmacological activity, fewer toxic side effects, and easy absorption ability (Li et al., 2017). Polysaccharides are also vital plant metabolites in traditional Chinese medicine, and more than 300 polysaccharide metabolites have been extracted from them (Luo et al., 2016). Traditional Chinese medicine plays an essential role in the treatment of diseases worldwide (Lu et al., 2022). The U.S. FDA has approved many plant-derived medicines. Therefore, studying the pharmacological activities of polysaccharides is of great significance for their development and application.

Many drugs to used treat UC have numerous side effects (Rahimi and Abdollahi, 2010; Tao and Siew, 2018). A natural product derived from traditional Chinese food and medicine has been developed as a vital complementary approach for ulcerative disease (Gilardi et al., 2014). Many reports suggest that polysaccharides have an anti-inflammatory effect in colitis mice (Zhang et al., 2020; Zou et al., 2020; Xiang et al., 2021; Yang et al., 2021). Polysaccharides are a vital metabolite of *Sonchus arvensis*. Our previous study confirmed that *Sonchus arvensis* L. extract had a protective effect in a UC model in mice, which may be related to gut microbiota diversity (Ren et al., 2023). Taken together, we hypothesized that *Sonchus arvensis* L. polysaccharide (SAP) may be an important metabolite of *Sonchus arvensis* L. extract that inhibits colitis and shapes the composition of the gut microbiota. Consequently, this study was designed to investigate whether polysaccharides can ameliorate colitis and explore the potential underlying molecular mechanisms.

## 2 Materials and methods

### 2.1 Materials

*Sonchus arvensis* L. was purchased from a local market. We verified the morphological characteristics of *Sonchus arvensis* L. using the pattern specimen. Our research team preserved the voucher specimens. DSS was purchased from MP Biomedicals (Irvine, CA). Millipore (Burlington, MA, United States) supplied all other chemicals.

### 2.2 Animals

*In vivo* experiments were performed in accordance with the ARRIVE guidelines. The Ethics Committee of Harbin Medical University and Heilongjiang Bayi Agricultural University approved the study protocol. Male C57BL/6 mice weighing 21 g–23 g were purchased from Charles River Laboratories (Beijing, China). A specific pathogen-free environment was provided for the house mice. During the investigation, no animals were sacrificed. The mice were euthanized by cervical dislocation at the end of the trial.

### 2.3 Preparation of the polysaccharide

Whole plants of *Sonchus arvensis L.* were cut into small pieces, which were soaked in 95% alcohol and then ultrasonicated for 0.5 h at 40 °C three times, and ethanol was removed in a ventilated kitchen. The pre-processed *Sonchus arvensis L.* sample was macerated for 2 hours with 23 mL of distilled water and then extracted for 1.8 h at 92 °C twice in a thermostatic water bath. The supernatant was collected and concentrated to 1 g/mL, anhydrous ethanol was then added four times, and the mixture was incubated at 4 °C overnight. The mixture was centrifuged, the supernatant was removed, and the precipitate was collected. Then, the precipitate was washed with anhydrous ethanol, acetone, and ether. The precipitate was dried in a drying cabinet at 40 °C and stored at −20 °C. The extraction yield was 10.13% ± 0.73%, and the total polysaccharide content in 100 mg of the extract was 35.27 ± 1.89 mg.

### 2.4 Establishment of the colitis model and treatment

**FIGURE 1:**
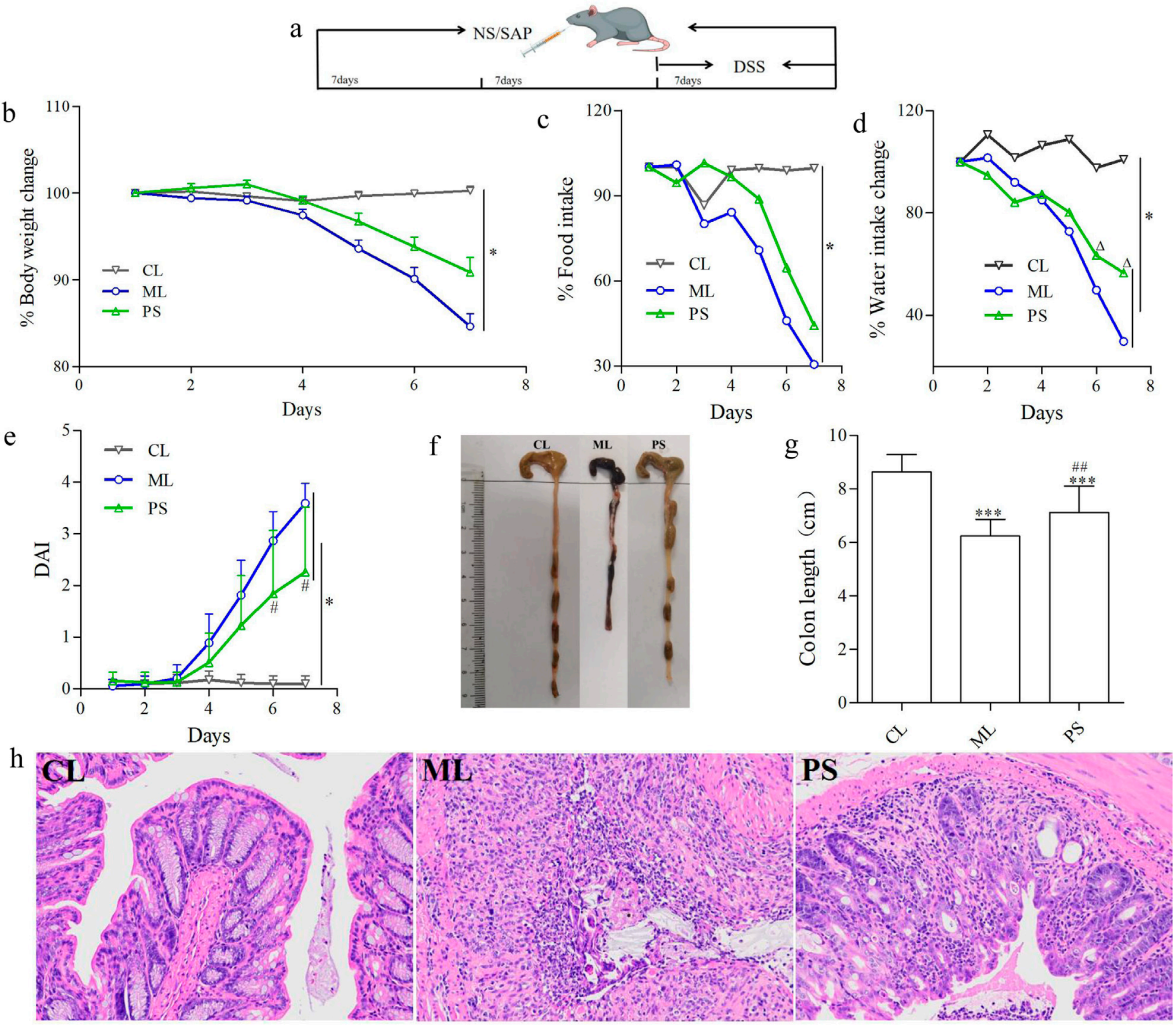
SAP decreased DSS-induced colitis in C57BL/6 mice. **(a)** Experimental design for evaluating the effects of SAP on DSS-induced colitis in mice. **(b)** Body weight change in each group. **(c)** Food intake change in each group. **(d)** Water intake change in each group. **(e)** Disease activity index (DAI). **(f**,**g)** Lengths of colons of mice in each group. **(h)** Colons from each experimental group were processed for histological evaluation (H&E staining ×200). Data were indicated as the means ± SD (n = 5) and were analyzed using the Student’s t-test; *p < 0. 05 and ***p < 0.001 vs. mice in the CL group, ^△^ *p* < 0.05 vs. mice in the ML group, ^##^ *p* < 0.05 vs. mice in the PS group.

An animal colitis model was established by intragastric administration of 0.2 mL of 0.6 g/mL DSS daily for seven consecutive days, as previously reported (Heng et al., 2017). All C57BL/6 mice were randomly grouped into three groups: the control group (CL), administered with physiological saline (NS) for 21 days continuously; the DSS model group (ML), administered with NS for 14 days and then treated with DSS for 7 days; and the SAP (1.26 g/kg) + DSS group (PS), treated with SAP for 21 days and then treated with SAP and DSS for 7 days. The SAP dosage was determined according to the previous study. Figure 1a shows the experimental timelines and grouping of all C57BL/6 mice.

### 2.5 Clinical scoring and histological analysis

The water intake, food intake, and body weight change of the animals were measured daily during the experiment. Diarrhea and rectal bleeding were monitored by observing the levels of water and blood in the feces. The disease activity index (DAI) was calculated based on previous reports (Ni et al., 2019). At the end of the experiment, all animals were sacrificed, and their colons were removed from the cecum to 1 cm above the anus. The colon length was measured, and the colon tissues were fixed in 4% paraformaldehyde. The hematoxylin and eosin (HE)-stained colon tissue sections were prepared by embedding colon tissue specimens in paraffin blocks, and the sections were observed using an optical microscope.

### 2.6 Immune organ index determination

At the end of the experiment, all the animals in all groups were weighed. The spleen and thymus were dissected, placed on filter paper to dry, and then weighed. The organ index formula is as follows:

Immune organ index (mg/g) = immune organ index (mg)/body weight (g).

### 2.7 ELISA test

At the end of the experiment, we collected blood samples from the retro-orbital sinus and allowed them to stand at room temperature for 2 h. The blood samples were centrifuged to obtain serum. IgA, IgG, and IgM levels were determined using ELISA kits.

### 2.8 Measurement of blood routine

At the end of the experiment, we collected blood samples from the retro-orbital sinus. Then, routine blood tests were performed using a BC-5500 fully automatic blood cell analyzer. During the measurements, red blood cells, platelets, white blood cells, and hemoglobin levels were recorded.

### 2.9 Gut microbiota analysis

We collected the cecal contents 7 days after DSS administration. Total genomic DNA was extracted from all samples using the CTAB method. Specific primers with barcodes were used to amplify the 16S rRNA genes from distinct regions. Then, we purified and quantified the products. The TruSeq® DNA PCR-Free Sample Preparation Kit was used to generate sequencing libraries, and index codes were added. Sequence analysis was performed using UPARSE software. The same OTUs were defined as those with ≥97% similarity. Further annotation was performed by screening a representative sequence from each OTU. A standard sequence number corresponding to the sample with the least number of sequences was used to normalize OTU abundance information. The output-normalized data were used to analyze alpha and beta diversity.

### 2.10 Non-targeted metabolomics analysis

We collected the cecal contents 7 days after DSS administration. Samples (50 mg) were added to 200 μL H_2_O and 800 µL MeOH/ACN, vortexed for 30 s, sonicated at 4 °C for 10 min, incubated at −20 °C for 60 min, and then centrifuged at 17,000 rpm. The supernatant was dried under vacuum, dissolved in 200 µL acetonitrile/water, vortexed, sonicated, and centrifuged, and the resulting supernatant was collected. A UHPLC-Q-TOF system was used to analyze the supernatant. An ACQUITY UPLC HSS T3 (1.8 μm; 2.1 × 100 mm) chromatographic column was used; the mobile phase was composed of A (0.1% formic acid in acetonitrile) and B (0.1% formic acid in water). The gradient elution was 5% A for 1 min, changing to 10% B within 1 min, then to 95% A within 12 min and held for 2 min, and finally to 5% A within 1 min and held for 3 min. The analytical column temperature was 35 °C, and the autosampler temperature was 4 °C. A 5 μL sample was used for each run.

Chromatographic data were analyzed using MS-DIAL 3.82 software. Key metabolites and metabolic pathways were identified using the KEGG, HMDB, MassBank, and METLIN databases.

### 2.11 Statistical analysis

Results are presented as the mean ± standard deviation (SD). GraphPad Prism was used to analyze the significance of the difference. *p* < 0.05 was considered statistically significant. QIIME software was used to analyze the alpha and beta diversity of microbiota. Progenesis QI V2.3 analyzed the LC–MS data. The groups in the data were analyzed by one-way ANOVA. Differential metabolite was selected with VIP >1.0 and *p* < 0.05.

## 3 Results

### 3.1 SAP attenuates DSS-induced colitis in C57BL/6 mice

The influence of SAP on UC was estimated using a DSS-induced colitis model in C57BL/6 mice. Body weight was significantly reduced in the ML and PS groups, especially in the ML group, compared to that of the control group (Figure 1b). However, SAP attenuated body weight loss compared to the ML group (Figure 1b). To determine whether the change in body weight was related to water or food intake, the average water and food intake were measured. The average water or food intake was slightly lower in the PS group than in the CL group (Figures 1c,d); however, it was clearly decreased in the ML group (Figures 1c,d). These findings suggest that SAP could markedly improve body weight loss in DSS-induced UC mice by attenuating appetite suppression.

The DAI displays the severity of the body weight change, diarrhea, and bloody stool. As shown in Figure 1e, the DAI scores of all groups showed a noticeable increase compared to those of the CL group; however, they were lower in the PS group than in the ML group (Figure 1e). Moreover, the colonic length was differently shortened in the ML group; nevertheless, this phenomenon was significantly ameliorated in the PS group (Figures 1f,g). HE analysis was used to further estimate the severity of colonic inflammation (Figure 1h). The ML group showed mucosal epithelial cell degeneration and necrosis, with a significant reduction in the number of intestinal glands, gland epithelial degeneration and shedding, interstitial edema, and extensive inflammatory cell infiltration. However, the pathological changes were significantly improved after SAP was administered to the DSS-induced UC mice. These results suggest that SAP may attenuate colitis in model mice.

### 3.2 SAP modifies immune parameters in mice with DSS-induced colitis

**FIGURE 2:**
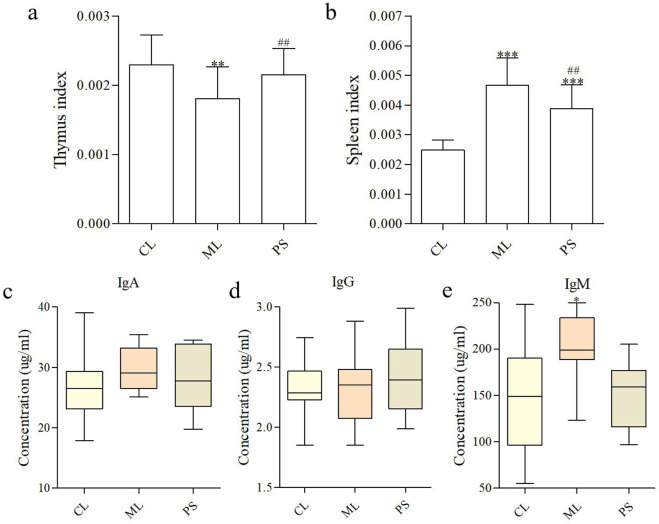
SAP modifies immune parameters. **(a**,**b)** Effects of SAP on the thymus index and spleen index. Effects of SAP on IgA **(c)**, IgM **(d)**, and IgG **(e)** in the serum of mice. Data were indicated as the means ± SD (n = 5) and were analyzed using the Student’s t-test; *p < 0. 05 and **p < 0.01 vs. mice in the CL group, ^##^ *p* < 0.05 vs. mice in the ML group.

The spleen index and thymus index reflect essential immune functions. Organ indices were calculated as the ratio of the organ to body weight. Figure 2a shows that the thymus index was not significantly different between the CL and PS groups; however, it increased dramatically in the ML group compared with those of the CL and PS groups. For spleen indices, although significant increases were observed in the ML and PS groups compared with those in the CL group, spleen indices decreased more in the PS group than in the ML group (Figure 2b).

B cells secrete immunoglobulins that participate in systemic humoral immunity. The major immunoglobulins are IgA, IgG, and IgM, which play an essential role in neutralizing pathogens and toxins. The effects of SAP on serum immunoglobulins are shown in Figures 2c–e. No significant difference in the IgA levels was observed between the groups. The IgM level was clearly higher in the ML group than in the CL and PS groups, indicating that DSS enhanced immunoglobulin secretion. The findings revealed that SAP plays a more effective role in immunoregulation by producing immunoglobulins.

We then determined the routine blood parameters in all groups, and the results are presented in Table 1. Almost all routine blood parameters showed statistically significant differences in the ML group compared with those in the CL group, whereas only a few parameters showed significant differences in the PS group (Neu%, Lym%, RBC, HCT%, HGB, and MPV). However, compared with those in the ML group, most parameters (Neu, Neu%, Lym%, RBC, HCT, MCV, RDW-SD, HGB, PLT, MPV, PDW, and PCT) were significantly different in the PS group. The findings suggest that SAP improved routine blood parameters in model mice.

**TABLE 1:** Levels of blood routine results in all the groups.

Detection index	CL	ML	PS
WBC (10^9^个/L)	6.99 ± 1.51	17.01 ± 13.31	6.98 ± 2.43
Neu (10^9^个/L)	1.02 ± 0.24	6.33 ± 4.81^**^	1.54 ± 0.50^#^
Neu% (%)	14.67 ± 2.59	39.83 ± 14.37^***^	21.96 ± 7.47^*##^
Lym (10^9^个/L)	5.96 ± 1.33	10.64 ± 8.96	5.43 ± 2.22
Lym% (%)	85.18 ± 2.64	59.88 ± 14.34^***^	79.39 ± 5.44^*###^
RBC (10^12^个/L)	10.14 ± 0.51	5.13 ± 2.67^***^	9.19 ± 0.61^*##^
HCT (%)	42.90 ± 1.94	22.84 ± 10.62^***^	39.15 ± 2.22^*##^
MCV (fL)	42.32 ± 0.67	45.90 ± 3.05^**^	42.66 ± 0.78^#^
RDW-CV	16.20 ± 1.23	17.14 ± 1.50*	15.53 ± 1.19
RDW-SD (fL)	29.63 ± 2.42	33.14 ± 4.50^**^	28.31 ± 2.28^#^
HGB (g/L)	143.60 ± 7.45	73.90 ± 36.01^***^	129.75 ± 7.77^*##^
MCH (pg)	14.18 ± 0.19	14.70 ± 0.72^*^	14.12 ± 0.23
MCHC (g/L)	334.50 ± 5.16	321.1 ± 16.39^*^	331.88 ± 3.14
PLT (10^9^个/L)	1162.8 ± 240.90	761.2 ± 277.44^***^	1237.9 ± 167.56^##^
MPV (fL)	5.04 ± 0.13	6.37 ± 0.77^***^	5.30 ± 0.11^**##^
PDW	14.92 ± 0.07	14.70 ± 0.09^***^	14.86 ± 0.10^##^
PCT (%)	0.59 ± 0.12	0.47 ± 0.13^**^	0.66 ± 0.09^##^

*p < 0.05, **p < 0.01, and ***p < 0.001 vs. CL; ^#^ *p* < 0.05, ^##^ *p* < 0.01, and ^###^ *p* < 0.001 vs. ML.

### 3.3 SAP ameliorates gut microbial dysbiosis in mice with DSS-induced colitis

**FIGURE 3:**
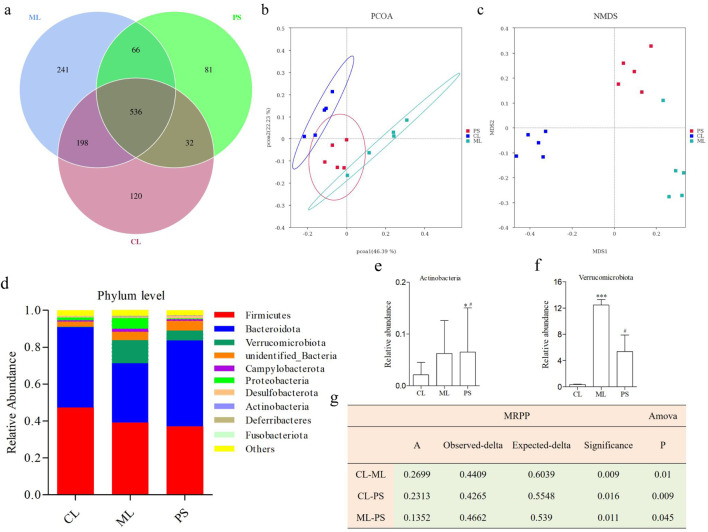
Effects of SAP on gut microbial dysbiosis. **(a)** Venn diagrams indicate the numbers of OTUs in the CL, ML, and PS groups. **(b)** Weighted UniFrac PCoA analysis of gut microbiota based on the OTU data of three groups. **(c)** Bray–Curtis NMDS analysis of gut microbiota based on the OTU data of three groups. **(d)** Relative abundance of gut microbiota from the cecal contents of all the groups at the phylum levels, classified by the representation of the top 10 species with the maximum abundance. Relative abundance of **(e)** Actinobacteria and **(f)** Verrucomicrobiota in cecal microbiota in the CL, ML, and PS groups. **(g)** Analysis of MRPP and AMOVA of gut microbiota among the CL, ML, and PS groups.

16S rRNA sequence analysis of the cecal samples was performed on the Illumina NovaSeq platform to evaluate the regulatory effect of PS on gut microbiota. The Venn diagram showed that 536 OTUs were shared among all the groups. In comparison, the CL and ML groups shared 734 OTUs, and the CL and PS groups shared 568 OTUs. The ML and PS groups shared 734 OTUs (Figure 3a). Furthermore, 120 unique OTUs were found in the CL group, 241 unique OTUs were found in the ML group, and 81 unique OTUs were found in the PS group (Figure 3a). According to the weighted UniFrac distance, PCoA showed different clustering of microbiota constituents among the CL, ML, and PS groups (Figure 3b). NMDS is a suitable ranking method for ecological research based on the Bray–Curtis distance. It also indicated distinct clustering of microbiota composition among the CL, ML, and PS groups (Figure 3c). The PCoA and NMDS results suggested that SAP induced a significant change in gut microbiota composition. As shown in Figure 3g, MRPP and ANOVA were utilized to detect further differences among the groups. The results of the MRPP and ANOVA analyses suggested that compositional variation in the GM among the three groups was significant (*p* < 0.05).

The structure of the bacterial community across the different groups was further assessed by analyzing the top 10 species with the highest phylum-level abundances. As shown in Figure 3d, the two most abundant phyla were Bacteroidetes and Firmicutes across all the groups. Relative abundances of Firmicutes and Bacteroidota were 47.28% ± 6.65% and 43.50% ± 7.22% in the CL group, 39.23% ± 13.05% and 32.00% ± 15.61% in the ML group, and 37.16% ± 7.59% and 46.49% ± 6.37% in the PS group, respectively. The following genera were identified: *Verrucomicrobiota*, *unidentified_Bacteria*, *Campylobacterota*, *Proteobacteria*, *Desulfobacterota*, *Actinobacteria*, *Deferribacteres*, and *Fusobacteriota*. It is worth noting that *Verrucomicrobiota* abundance was significantly increased in the model group. However, this increase was remarkably reversed after SAP treatment (Figure 3f). Moreover, *Actinobacteria* abundance was significantly higher in the PS group (Figure 3e). Overall, these findings indicate that SAP can alter GM abundance.

**FIGURE 4:**
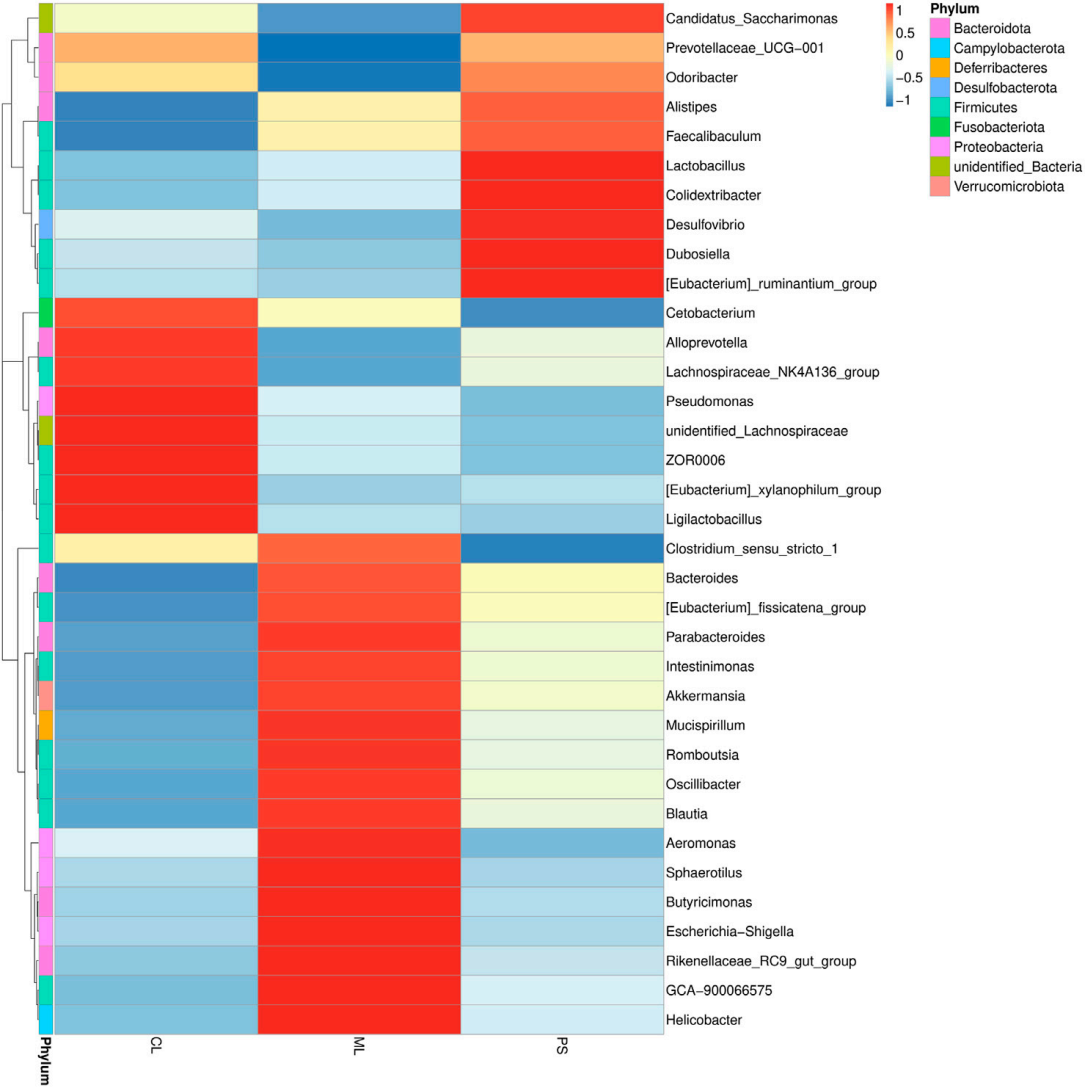
Heatmap of microbial distributions at genus level in the CL, ML, and PS groups. Blocks in red and blue indicate the high and low z-score values of OUT sizes, respectively.

We further analyzed the degree of taxonomic similarity among the different groups at the genus level. As shown in Figure 4, the taxa heatmap indicated that the CL and SP groups clustered well at the genus level and were clearly separated from the ML group. This suggests that the gut microbiota composition of mice in the PS group was closer to that of the CL group.

**FIGURE 5:**
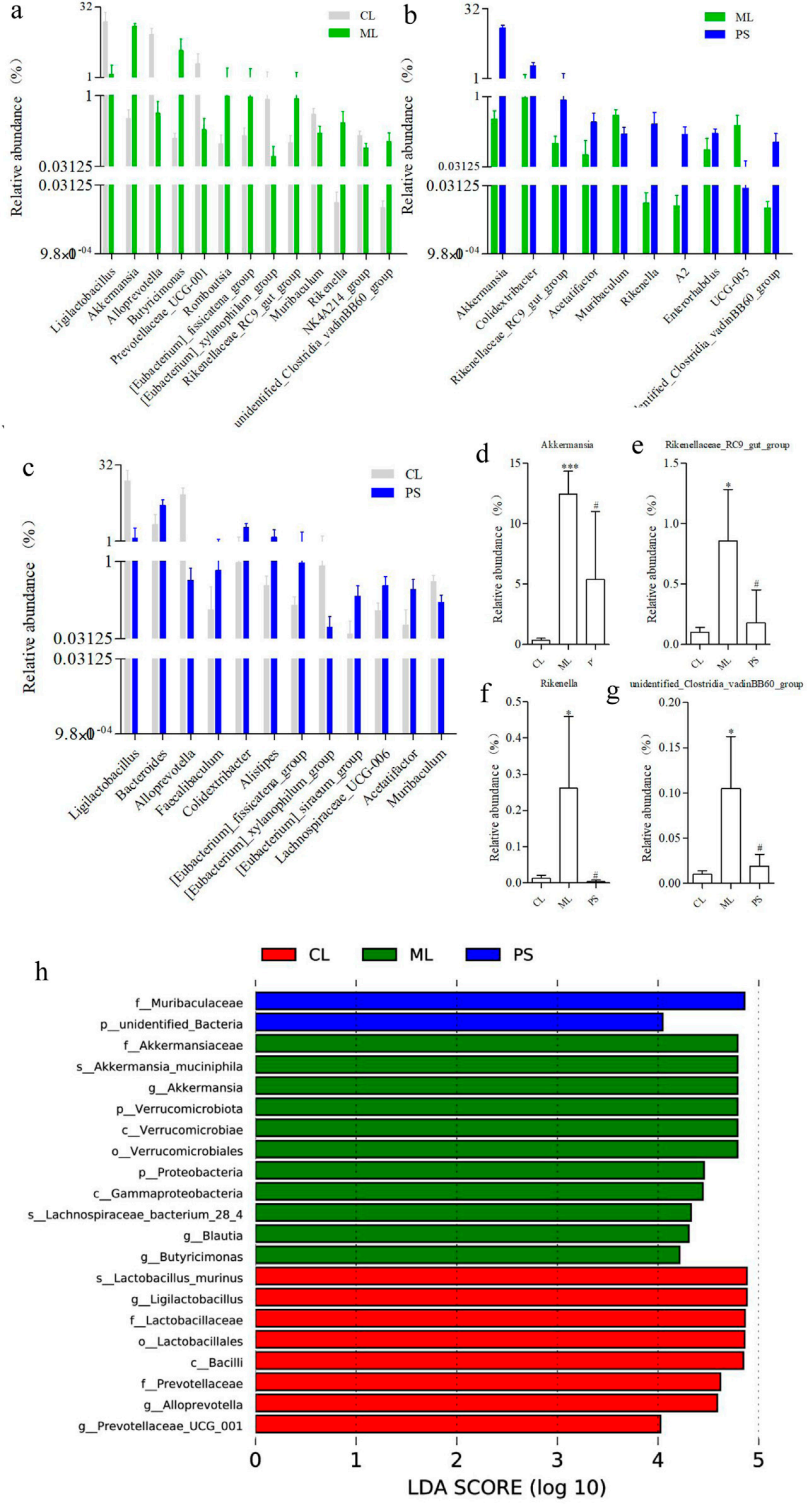
Categories of species that are obviously different between the two groups at the genus level: **(a)** CL group vs. ML group, **(b)** CL group vs. PS group, and **(c)** ML group vs. PS group. Relative abundance of **(d)** Akkermansia, **(e)** Rikenellaceae_RC9_gut_group, **(f)** Rikenella, and **(g)** unidentified_Clostridia_vadinBB60_group in cecal microbiota in the CL, ML, and PS groups. **(h)** LEfSe analysis of the gut microbiota differed among CL, ML, and PS groups. The statistical test was performed using the LDA effect size method.

To search for differences across species, we also conducted t-tests at the genus level. Figures 5a–c show the differentially exhibited microbiota between the groups at the genus level. It is worth noting that there were no significant differences in the abundances of *Akkermansia*, *Rikenella*, *Rikenellaceae_RC9_gut_group*, and *unidentified_Clostridia_vadinBB60_group* between the CL and PS groups. In contrast, their abundance was clearly increased in model mice; however, this alteration was remarkably reversed after PS administration (Figures 5d–g). They were attached to three phyla (Verrucomicrobiota, Bacteroidota, and Firmicutes), three classes (Verrucomicrobiae, Bacteroidia, and Clostridia), three orders (Verrucomicrobiales, Bacteroidales, and Clostridia_vadinBB60_group), three families (Akkermansiaceae, Rikenellaceae, and unidentified Clostridia_vadinBB60_group), and four genus levels. We also performed LEfSe analysis to determine the detailed composition of the GM. As indicated in Figure 5h, compared with the CL group, the Verrucomicrobiota phylum, Verrucomicrobiae class, Verrucomicrobiales order, Akkermansiaceae family, and Akkermansia genus were significant biomarkers after DSS-induced UC in mice; however, the biomarker levels changed after SAP treatment, which was similar to the results of the T-test. Overall, DSS induced microbial dysbiosis in mice, whereas SAP could regulate GM dysbiosis to restore intestinal homeostasis.

### 3.4 SAP regulates metabolites in mice with DSS-induced colitis

**FIGURE 6:**
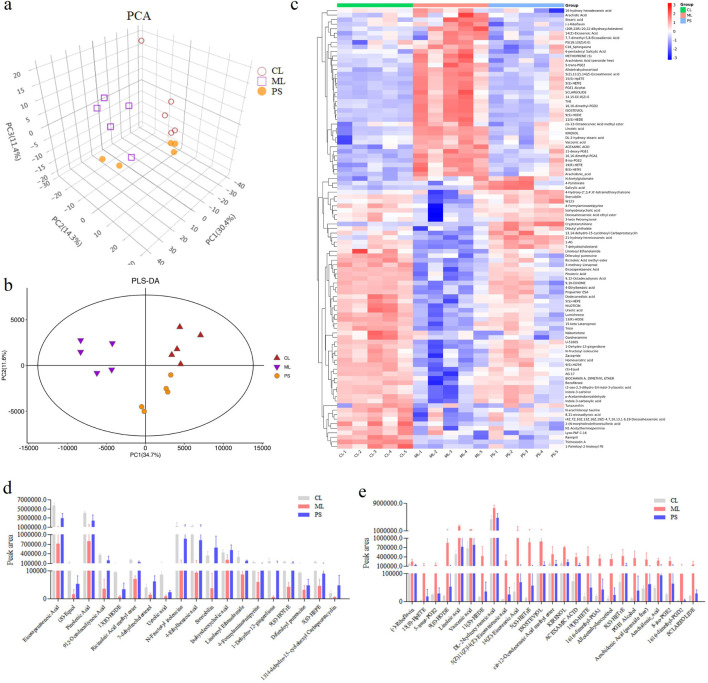
Effects of SAP on the metabolites. **(a)** Principal component analysis (PCA) plot showing clustering between the groups. **(b)** PLS-DA model predicting the differences between the samples. **(c)** Differential metabolites analysis by heatmap in the CL, ML, and PS groups. **(d**,**e)** Differences in the metabolites amount; the amount of all the metabolites is significantly different between the CL and ML groups (*p* < 0.05).

The gut microbiota is involved in host metabolism and, in turn, affects metabolite production. The effects of SAP on metabolites were studied using metabolomics. UPLC-QTOF-MS/MS was used to detect 1,219 metabolites in the cecal contents of mice. As indicated in Figures 6a,b, clear metabolic differentiation was observed among the groups. There were 97 different metabolites in the groups (Figure 6c). Further data analysis found that the levels of the 19 metabolites was decreased in the ML group compared to those in the CL group, while SAP treatment could restore these changes (Figure 6d); meanwhile, compared with the CL group, DSS administration increased the amount of 25 metabolites, while these changes were remarkably reversed after PS treatment (Figure 6e). Overall, these findings displayed that the effect of SAP on UC is mediated by metabolic modification.

**FIGURE 7:**
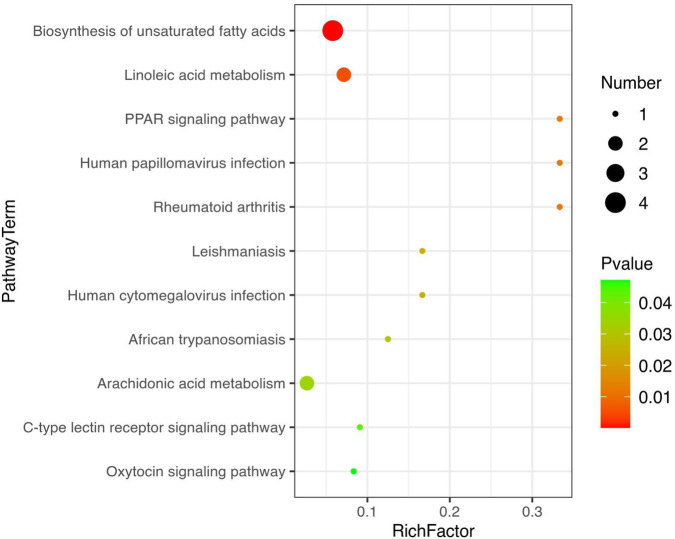
Metabolic pathway enrichment analysis was performed for the differential metabolites by the KEGG database.

Metabolic pathway enrichment analysis was carried out for differentially expressed metabolites using the KEGG database. The linoleic acid metabolism and the biosynthesis of the unsaturated fatty acids pathway were mainly affected after SAP treatment compared with other metabolic pathways (Figure 7). In these two metabolic pathways, the levels of the four metabolites (linoleic acid, DL-2-hydroxy stearic acid, arachidonic acid, and 9(S)-HODE) were distinctly increased in the colitis model mice compared to those in the CL group, and SAP recovered the levels of these four metabolites (Figure 6e). Moreover, in the linoleic acid metabolism pathway, the level of the metabolite (13(R)-HODE) was obviously decreased in the ML group compared to that in the CL group. At the same time, this alteration was remarkably reversed after PS treatment (Figure 6d).

### 3.5 Correlation analysis of gut microbiota and metabolites

**FIGURE 8:**
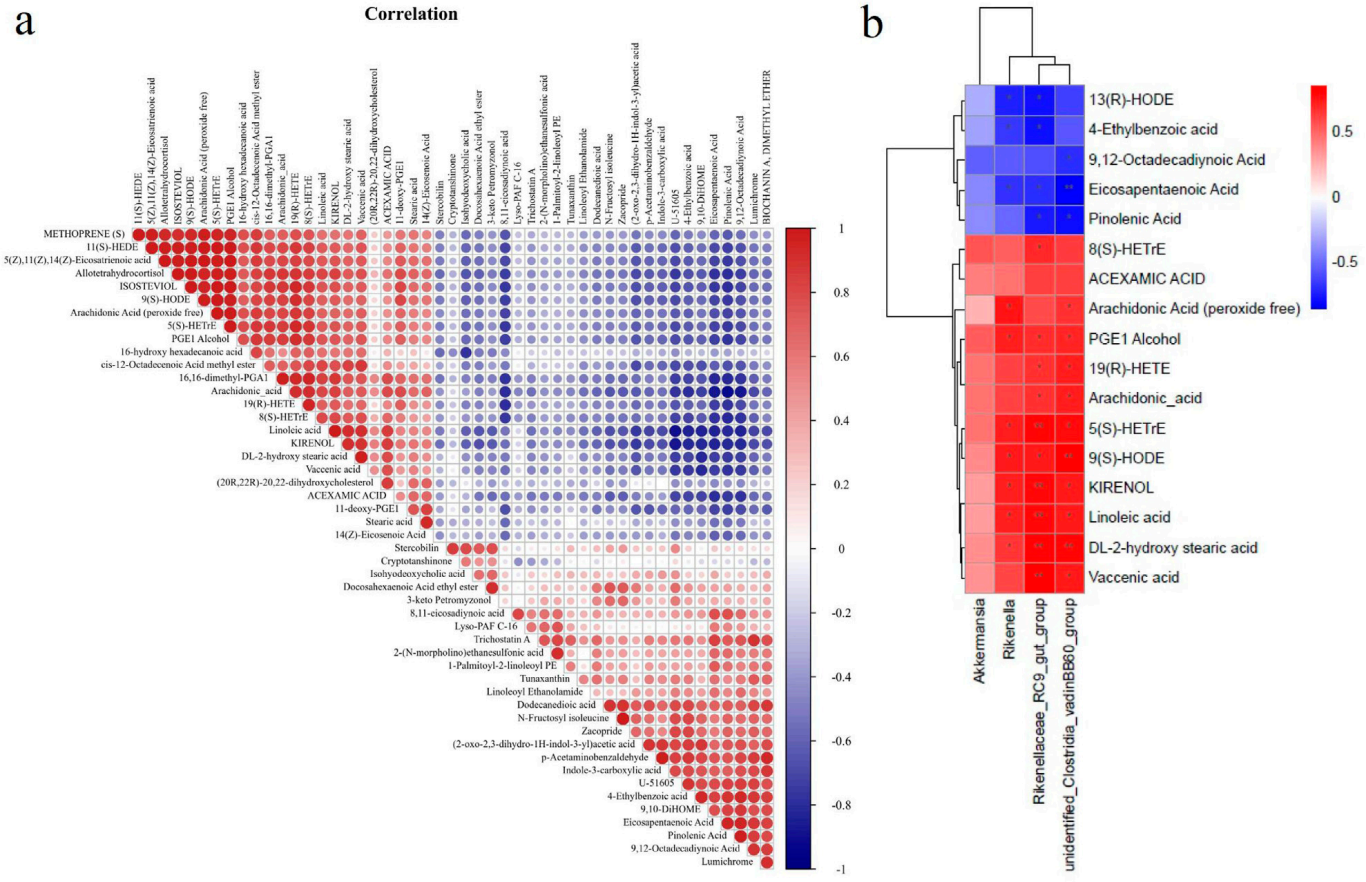
Correlation analysis of gut microbiota and metabolites. **(a)** Pearson’s rank correlation analysis on altered metabolites to investigate the effects of SAP on metabolites. **(b)** Spearman’s rank correlation analysis detecting the association between the 4 altered gut microbiota and 17 altered metabolites. Red and blue indicate the positive and negative correlations, respectively, and *p < 0.05 and **p < 0.01.

We conducted Pearson’s rank correlation analysis on the altered metabolites to investigate the influence of SAP on metabolites (Figure 8a). The findings displayed that 10 metabolites were significantly correlated with linoleic acid, and four metabolites (kirenol, DL-2-hydroxy stearic acid, vaccenic acid, and acexamic acid) were significantly positively correlated with linoleic acid. In comparison, six metabolites (U-51605, 4-ethylbenzoic acid, 9,10-DiHOME, eicosapentaenoic acid, pinolenic acid, and 9,12-octadecadiynoic acid) showed a clear negative correlation with linoleic acid (Figure 8a). For DL-2-hydroxy stearic acid, the related metabolites were similar to those for linoleic acid, except for kirenol. Our findings demonstrated that nine metabolites were clearly correlated with 9(S)-HODE; three metabolites (arachidonic acid (peroxide free), 5(S)-HETrE, and PGE1 alcohol) were significantly positively correlated with 9(S)-HODE, while six metabolites (8,11-eicosadiynoic acid, (2-oxo-2,3-dihydro-1H-indol-3-yl) acetic acid, U-51605, eicosapentaenoic acid, pinolenic acid, and 9,12-octadecadiynoic acid) displayed a significant negative correlation with 9(S)-HODE (Figure 8a). For arachidonic acid, two metabolites (19(R)-HETE) and 8(S)-HETrE) were positively correlated, whereas six metabolites (8,11-eicosadiynoic acid, U-51605, 4-ethylbenzoic acid, eicosapentaenoic acid, pinolenic acid, and 9,12-octadecadiynoic acid) showed a markedly negative correlation (Figure 8a). However, as shown in Figure 8a, no metabolites correlated with 13(R)-HODE.

Spearman’s rank correlation analysis was performed to examine the relationships between the 4 altered GM and 17 altered metabolites, clarifying how changes in GM abundance are associated with the altered metabolites (Figure 8b). The findings revealed that three altered GM taxa showed a clear positive or negative correlation with 16 altered metabolites (Figure 8b). The relative abundance of Rikenella was positively correlated with seven metabolites (arachidonic acid (peroxide free), PGE1 alcohol, 5(S)-HETrE, 9(S)-HODE, kirenol, linoleic acid, and DL-2-hydroxy stearic acid) and negatively correlated with three metabolites (13(R)-HODE, 4-ethylbenzoic acid, and eicosapentaenoic acid). For the relative abundance of Rikenellaceae_RC9_gut_group, it showed a positive correlation with the contents of 10 metabolites (8(S)-HETrE, PGE1 alcohol, 19(R)-HETE, arachidonic acid, 5(S)-HETrE, 9(S)-HODE, kirenol, linoleic acid, DL-2-hydroxy stearic acid, and vaccenic acid) and a negative correlation with the contents of four metabolites (13(R)-HODE, 4-ethylbenzoic acid, pinolenic acid, and eicosapentaenoic acid). Moreover, unidentified_Clostridia_vadinBB60_group abundance was positively correlated with 10 metabolites (arachidonic acid (peroxide free), PGE1 alcohol, 19(R)-HETE, arachidonic acid, 5(S)-HETrE, 9(S)-HODE, kirenol, linoleic acid, DL-2-hydroxy stearic acid, and vaccenic acid). It was negatively associated with three metabolites (9,12-octadecadiynoic acid, eicosapentaenoic acid, and pinolenic acid).

## 4 Discussion

Ulcerative disease is a chronic inflammatory condition (Khor et al., 2011) that severely affects patients’ daily lives, studies, and work (Guindi and Riddell, 2004). However, many drugs used to treat UC have numerous side effects (Rahimi and Abdollahi, 2010; Tao and Siew, 2018). A natural product derived from traditional Chinese food and medicine has been developed as a vital complementary approach for ulcerative disease (Gilardi et al., 2014). In the present study, SAP, the polysaccharide from *Sonchus arvensis* L., effectively attenuated DSS-induced colitis, as demonstrated by body weight changes, DAI, and HE in model mice after 1 week of treatment. Yang et al. (2021) reported that Fuzhuan brick tea polysaccharide improved colitis, *Mytilus coruscus* polysaccharide has an anti-inflammatory effect in colitis mice (Xiang et al., 2021), *Ficus carica* polysaccharide attenuates DSS-induced colitis in C57BL/6 mice (Zou et al., 2020), and polysaccharides from *Flammulina velutipes* improve colitis (Zhang et al., 2020). Our findings are similar to those of the studies mentioned above. Because our research is an early-stage, single-dose study, the 4R rules (reduce, refine, replace, and responsibility) are used.

Polysaccharides play a crucial pharmacological role in non-specific and specific immunity (Shao et al., 2006). It can activate immune cells, increase antibody levels, promote cytokine release, and activate the complement system (Schepetkin et al., 2013; Sheu et al., 2013). The spleen index and thymus index reflect essential immune functions. Therefore, we first determined the spleen index and thymus index, and the results showed that SAP reversed thymic atrophy induced by DSS. This finding is consistent with those of previous reports (Wang et al., 2011). Immunoglobulins (IgGs) are involved in systemic humoral immunity. The major immunoglobulins are IgA, IgG, and IgM, and they play an essential role in neutralizing pathogens and toxins, making pathogens more susceptible to phagocytosis by various phagocytic cells (Ollila and Vihinen, 2005). Our results showed that SAP played a more effective role in immune regulation, as evidenced by its effect on immunoglobulin production.

Neutrophils are the most abundant immune cells in the body and play a vital role in the immune response. Neutrophils recognize molecular receptors on the microbial surface at the site of infection, activate signaling pathways, ultimately prolong cell survival, promote adhesion and phagocytosis, and enhance cytokine production, which supports the immune response (Lauvau et al., 2014). Lymphocytes participate in immune reactions through cellular and humoral regulation. It was found that patients with UC had altered lymphocytes (Jorgensen and Miao, 2015). Platelets can promote hemostasis, repair endothelial tissue, and participate in the body’s inflammatory response. In the presence of inflammation, platelet counts can change, and some inflammatory diseases use PLR as a clinical indicator of disease severity (Park et al., 2013; Do Carmo et al., 2014). Anemia is a common complication of inflammatory bowel disease that responds to disease severity to some extent. The most common anemia in IBD is iron deficiency anemia (Gomollon and Gisbert, 2009; Oustamanolakis et al., 2011), which is associated with PLT. Therefore, routine blood parameters were determined for all groups. As shown in Table 1, SAP reversed most routine blood parameters in the UC model mice.

The gut microbiota is a micro-ecosystem that parasitizes the human body and plays a vital role in maintaining human health (Nishida et al., 2018). It has been reported that the composition of gut microbiota is altered in patients with inflammatory bowel diseases compared to that in healthy humans (Sartor and Wu, 2017; Takahashi et al., 2016). Therefore, GM is a possible target for UC treatment. Exposure to intestinal microbes is unavoidable after the oral administration of traditional Chinese medicine. It is well-known that digestion in the stomach or small intestine does not degrade or absorb natural polysaccharides (EI Kaoutari et al., 2013). However, polysaccharides can be metabolized by the microflora in the large intestine to exert their physiological functions. Thus, we hypothesized that the intestinal microflora may be responsible for SAP’s anti-inflammatory effect. Therefore, 16S rRNA amplicon sequencing was performed to investigate whether SAP could regulate gut microbiota dysbiosis in UC and restore intestinal homeostasis. Our study indicated that SAP treatment remarkably reversed the increase in the abundance of *Akkermansia*, *Rikenella*, *Rikenellaceae_RC9_gut_group*, and *unidentified_Clostridia_vadinBB60_group* in DSS-induced UC mice (Figure 5). This finding is similar to those of previous reports (Guo et al., 2020), which suggest that regulating these bacterial communities may be a common characteristic of polysaccharides exerting probiotic effects.

Compared to other metabolic pathways, SAP treatment mainly affected linoleic acid metabolism and the biosynthesis of unsaturated fatty acids (Figure 7). The biosynthesis of unsaturated fatty acids is mediated by arachidonic acid (Lillo-Carmona et al., 2020). In this study, many arachidic acid derivatives were found to be important for SAP treatment of UC. In addition, these metabolites are associated with perturbations in linoleic acid metabolism. These metabolite changes may alter linoleic acid metabolism in the host. It has been reported that the disease is caused by perturbation of linoleic acid metabolism (Li et al., 2017). The three polyunsaturated fatty acids (PUFAs), namely, eicosapentaenoic acid, pinolenic acid, and 9,12-octadecadiynoic acid, have anti-inflammatory effects (Richards et al., 2020; Wu et al., 2019), and humans need to consume PUFAs, which are indispensable for cell survival (Lee et al., 2019; Stoffel et al., 2020). 4-Ethylbenzoic acid is a phenolic acid produced during colon fermentation that regulates inflammatory signaling molecules (Bessonneau et al., 2021). 13(R)-HODE inhibits IL-6 release from monocytes, which suggests that the lipids may play a vital role in controlling inflammatory responses (Rolin et al., 2014). In summary, Figures 5d–g, 6d,e, 8 show that five metabolites (eicosapentaenoic acid, pinolenic acid, 9,12-octadecadiynoic acid, 4-ethylbenzoic acid, and 13(R)-HODE) were key to UC treatment, and SAP could regulate these five metabolites.

Despite the promising results, several limitations of this study should be acknowledged. First, the DSS-induced colitis model, while well-established, primarily represents an acute injury and repair mechanism. It may not fully recapitulate the chronic, relapsing nature of human ulcerative colitis, which involves complex, dysregulated immune responses over a long period. Therefore, the therapeutic efficacy of SAP should be further validated in chronic colitis models or other genetic models to better assess its long-term benefits and potential to maintain remission.

## 5 Conclusion

SAP improved the DAI score and colonic length in DSS-induced colitis in mice and reduced pathologic damage, suggesting that SAP has a protective effect in this model. Moreover, SAP treatment modified immune parameters in colitis mice. 16S rRNA sequencing revealed that the abundance of Akkermansia, Rikenella, Rikenellaceae_RC9_gut_group, and unidentified_Clostridia_vadinBB60_group in the UC model mice was remarkably reversed after PS treatment. The results of the correlation analysis of GM and the metabolites demonstrated that SAP could regulate five metabolites (eicosapentaenoic acid, pinolenic acid, 9,12-octadecadiynoic acid, 4-ethylbenzoic acid, and 13(R)-HODE), which are correlated with GM. The protective effect of SAP on the model mice may be related to GM diversity and metabolites.

## Data Availability

The original contributions presented in the study are publicly available. This data can be found at the NCBI repository, accession number PRJNA1391682, available at: https://www.ncbi.nlm.nih.gov/sra/PRJNA1391682.
